# Systematic review of implementing stewardship activities to promote appropriate antimicrobial use

**DOI:** 10.1017/ash.2026.10769

**Published:** 2026-08-03

**Authors:** Virginia McKay, Emmanuel Tetteh, Kelly Bono, Collin McGovern, Mathew Sattler, Luke Zabotka, Lauren Yaeger, Harry Obeng, Joshua M. Perez-Cruet, Evan Facer, Jason G. Newland, Sara Malone

**Affiliations:** 1 Washington University In St Louis, USA; 2 Washington University School of Medicine in Saint Louis, USA; 3 Nationwide Children‘s Hospital, USA

## Abstract

**Objective:**

This systematic review aims to describe the impact of specific antimicrobial stewardship program (ASP) strategies on implementation and stewardship-related outcomes.

**Design:**

Systematic literature review.

**Methods:**

We systematically searched multiple databases using search terms that focused on stewardship and implementation. An original search was completed on October 29, 2021, and then updated on February 26, 2025. Studies were eligible for inclusion if they were in English, reported an antimicrobial resource outcome, and using either a comparison group or a pre/post design as a minimum level of rigor. Data from articles were extracted to understand the context and design of the evaluation, the types of approaches used, and outcomes assessed.

**Results:**

Out of 2035 studies that were identified, we included 250 full-text peer-reviewed manuscripts. Of the 250 studies included, 52% were conducted in the USA. The majority (80%) of studies were conducted in inpatient settings, particularly in adult academic hospitals (29%). Programs were often implemented hospital-wide or across multiple specialties, with a focus on emergency medicine and intensive care units. Education or learning (64% of studies) and handshake stewardship (56% of studies) were by far the most common ASP strategies used. Mortality (36% of studies) and drug choice (57% of studies) were the most tracked patient and resource outcomes, respectively.

**Conclusions:**

Our review highlights the variability in approaches as part of ASPs, indicating a need for a more systematic understanding of how different approaches are implemented and their subsequent impact on antimicrobial use.

## Introduction

The rapid rise of antimicrobial resistant organisms (AMR) has severely impacted healthcare costs and mortality rates.^1,2^ In response to this crisis, the United Nations (UN), World Health Organization (WHO), and the Centers for Disease Control and Prevention (CDC) have identified the development of antimicrobial stewardship programs (ASPs) as critical tools to combat this escalating problem in global health.^3,4^ Generally, ASPs encompass a set of directed activities or strategies designed to optimize antimicrobial use without compromising patient health outcomes.^5,6^ ASPs then critically examine the appropriate antimicrobial agent, dose, route of administration, and therapy duration to determine whether strategies were successful.^5^ Toward this end, the CDC has developed core strategies for ASPs in both the inpatient and outpatient settings.^7^

Systematic reviews indicate that ASPs are effective and necessary to impact antimicrobial optimization;^6,8,9^ highlighting many potential strategies that programs might employ, stewardship-related outcomes, and patient-level outcomes. However, to date, reviews have not examined the effectiveness of specific strategies. For example, audit and feedback as a strategy is used routinely in stewardship, but it is currently unclear the extent to which this strategy is consistently effective or if this strategy is effective in specific circumstances. Synthesis of this literature is needed to guide ASP program implementation when resources may be limited and stewardship teams must make the most judicious use of their efforts in healthcare settings.^6,10,11^

Implementation science is a burgeoning field that works to create generalizable knowledge about strategies to adopt and integrate evidence-based health interventions into clinical and community settings for maximal population benefit.^12^ This includes approaches frequently leveraged by ASP teams targeting clinicians such as education, audit and feedback, or automated reminders. A key potential benefit of implementation science for stewardship is the specification of these strategies and expected intermediate outcomes of strategies, termed implementation outcomes.^13^ These include outcomes that one would expect to be immediately impacted by the strategy such as increased knowledge, more frequent acceptance of stewardship recommendations, and equitable reach of strategies and impact among eligible clinicians. This level of specificity and rigor in revealing the underlying mechanisms that make implementation strategies, like audit and feedback, effective has the potential to refine stewardship efforts.

The purpose of this systematic review is to describe the impact of specific stewardship strategies on stewardship-related outcomes while also considering implementation outcomes that may mediate the success of stewardship initiatives.

## Methods

We followed PRISMA (Preferred Reporting Items for Systematic Reviews and Meta-Analyses) guidelines to guide our literature search, inclusion/exclusion process, and data extraction (Supplemental File 1).^14^

### Search methodology

A medical librarian (LHY) searched the literature for records including the concepts of implementation science, antimicrobial stewardship, and outcomes. The librarian created search strategies using a combination of keywords and controlled vocabulary in Embase.com 1947-, Ovid Medline 1946-, Scopus 1823-, The Cochrane Database of Systematic Reviews (CDSR), Cochrane Central Register of Controlled Trials (CENTRAL), The Cumulative Index to Nursing and Allied Health Literature (CINAHL Plus) 1937-, Antimicrobial Stewardship and Healthcare Epidemiology (ASHE), and Clinicaltrials.gov 1997-. Our initial search strategies were completed on October 29, 2021, and a total of 1,779 results were found. Our search was updated on February 26, 2025, to include more recent publications on the topic and yielded 989 additional publications. Both searches yielded a total of 2,035 records, of which 17 duplicate records were deleted after using the de-duplication processes described in “De-duplication of database search results for systematic reviews in EndNote”.^15^ Fully reproducible search strategies for each database can be found in the Supplemental File 2.

### Inclusion/exclusion criteria

Articles were included if they met the following criteria: article was a full-length, peer-reviewed manuscript written in English or translated to English; research intended to impact antimicrobial use with humans with a reported resource utilization outcome (ie, outcomes related to the use of hospital and material resources, such as duration of therapy and associated costs) using either a comparison group or pre/post design. Articles were excluded if they were: Conference or poster abstracts; research with animals or not having an antimicrobial impact; did not describe specific ASP strategies; only provided descriptive outcomes, only described barriers or facilitators to stewardship, were post evaluation only, or did not have a comparison group.

### Extraction

Two reviewers extracted data from each paper, each working independently and then extraction results were compared. Any extraction conflicts were discussed between the reviewers and resolved, using a third team member if necessary. The data extraction form used to collect variables across all included studies is provided in Supplemental File 3.

Supplemental File 4 includes all the variables extracted including categories and a brief description. Briefly, variables fell into five broad categories: article identifiers (author, year, title, journal), description of the research context (country, healthcare setting, medical specialty, disease targeted, patient population, etc.), elements of the research design (stated design, pre- and pos-tintervention data collection period, sample size, use of theory, etc.), the ASP strategies leveraged (ASP strategy used, level of ASP team involvement, focus of strategy, etc.), and outcomes reported (implementation science outcomes, resource utilization outcomes, and primary patient outcomes). For strategies, we relied on how authors named the strategy. A selection of key variables and definitions are provided in Table 1.

**Table 1.** Key outcome domains and definitions Table 1 long description.

Outcome type	Definition
***Implementation outcomes***	
Knowledge	Extent to which providers understand the intervention or prescribing practice being implemented.
Awareness	Whether clinicians are aware of protocol or guideline changes, distinct from deeper knowledge.
Acceptability	Extent to which an intervention or strategy is perceived as satisfactory.
Feasibility	Provider perception of the practicality of the intervention in their clinical setting.
Appropriateness	Provider perception of how compatible the intervention is with their context or patient population.
Fidelity	Degree to which providers adhere to guidelines or protocols as intended.
Reach	Proportion of the intended audience or setting exposed to or engaged with the strategy.
Adaptation	Modifications made to the intervention to accommodate local context.
Sustainment	Continuation of the intervention beyond the active study period.
Implementation Cost	Financial or non-financial resources required to implement the intervention (eg, staff time).
***Resource utilization outcomes***	
Overall consumption	Total antibiotic use at the unit or institutional level (eg, days of therapy, defined daily doses per 1,000 patient-days).
Drug choice	Changes in the antimicrobial agent selected toward more appropriate or narrower-spectrum therapy.
Duration	Length of antimicrobial treatment course.
Dose	Quantity of antibiotic administered per treatment case.
IV-to-oral switch	Conversion from intravenous to oral antimicrobial therapy.
Timing	Appropriateness of when therapy is initiated (eg, time from diagnosis to first dose).
Diagnostic switch	Conversion to more targeted or more effective diagnostics.
Resource Cost	Financial costs associated with antimicrobial prescribing or administration.
***Primary patient outcomes***	
Mortality	All-cause or infection-related death during the study period.
Length of stay	Days hospitalized, used as a proxy for illness severity and recovery.
Infections	Incidence of CDI, SSI, or other infections arising from antimicrobial use or reduction.
Admissions/readmissions	Hospital admission or 30-day readmission rates.
MDRO emergence	Development or spread of multi-drug-resistant organisms within the institution.
***Outcome effect classification***	
All beneficial	All outcomes in a category showed a statistically significant or clinically meaningful improvement.
All harmful	All outcomes showed statistically significant or clinically meaningful worsening.
Mixed	A combination of beneficial, harmful, or null effects reported within a category.
Null	No statistically significant or clinically meaningful change in either direction.

### Data management and analysis

Data were managed and analyzed in R. Data were reviewed for missingness, and variable categories were collapsed as appropriate. Outcomes were explored as descriptive statistics.

## Results

**Figure 1.**
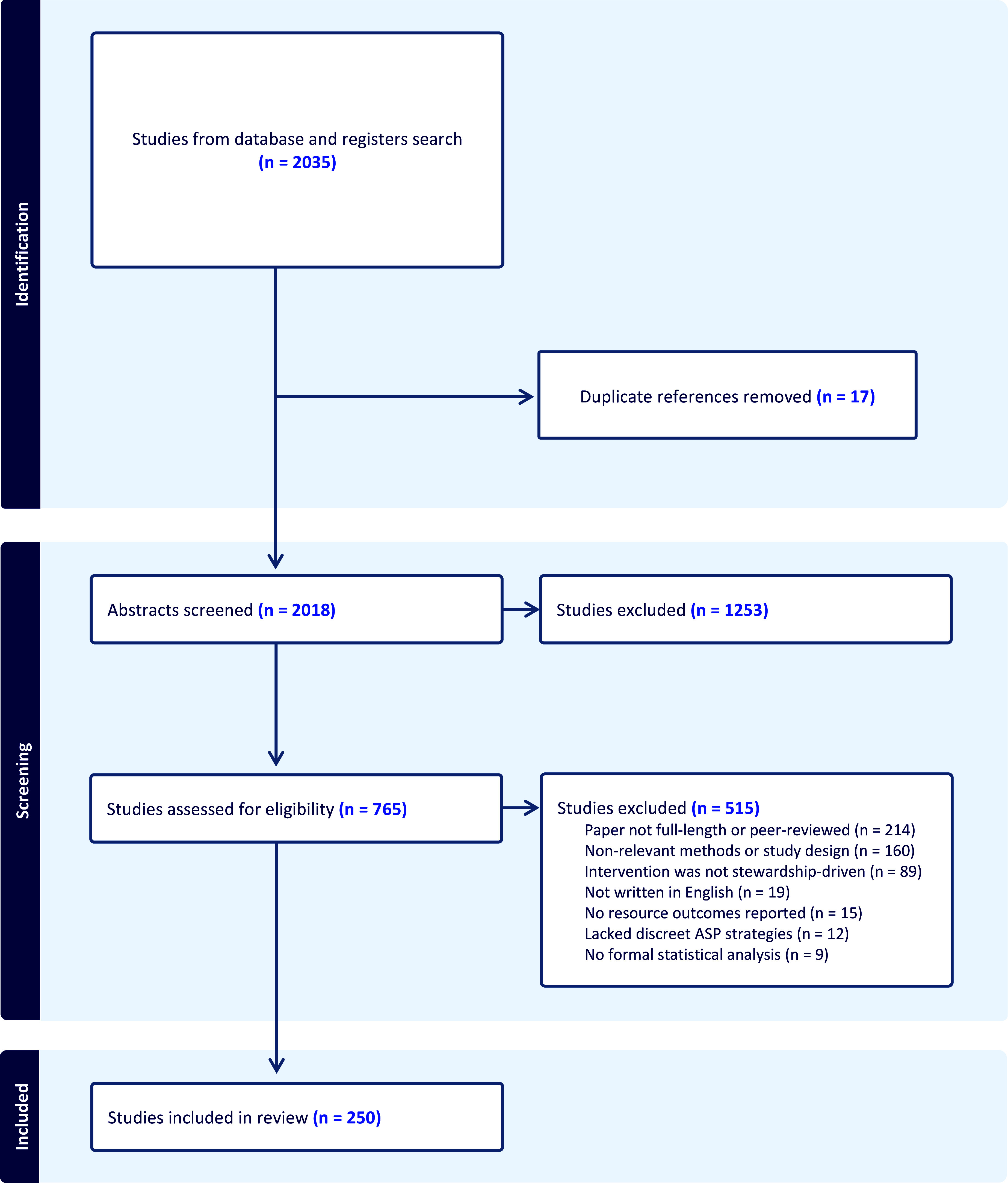
PRISMA flow chart.

Figure 1 is a flow chart describing citations and articles included. The initial database yielded 2,035 potential citations. Seventeen citations were duplicates and removed. During abstract screening 1,253 citations were excluded because they were not full-length articles, did not focus on humans, were not in English, or we could not obtain the full-length article. During full text screening, another 515 articles were excluded because the research did not include the appropriate research design elements, have a stewardship-related outcome, did not describe specific stewardship strategies in addition to criteria described above. A total of 250 full-length articles were reviewed and included in this study.

### Characteristics of included studies

Table 2 shows the characteristics of articles that were included in this review. Most studies were conducted in the United States (51.6%). The vast majority of studies were conducted (80.2%) in inpatient settings. Among studies conducted in inpatient settings, the majority were conducted in adult academic or teaching hospitals (30.0%) and community hospitals (22.3%). Adults also constituted the majority of patient populations where ASPs were conducted (75.3%).

**Table 2.** Characteristics of included studies (N = 250) Table 2 long description.

Characteristic	n (%)
**Region**	
USA	129 (51.6)
European Region	37 (14.8)
Western Pacific	32 (12.8)
Canada	18 (5.2)
Eastern Mediterranean	13 (7.2)
South-East Asia Region	10 (4.0)
Region of the Americas (excl. USA and Canada)	5 (2.0)
African region	3 (1.2)
Not specified	3 (1.2)
**Settings**	
Inpatient	198 (80.2)
Outpatient	27 (10.9)
Both inpatient and outpatient	19 (7.7)
**Settings**	
Academic or teaching adult hospital	74 (30.0)
Community hospital	55 (22.3)
Multiple inpatient settings	14 (5.7)
Academic or teaching children‘s hospital	17 (6.9)
Veterans hospital	14 (5.7)
Long-term care and nursing homes	11 (4.5)
Not specified	44 (17.8)
**Age of patient population**	
Adult only	186 (75.3)
Pediatrics only	32 (13.0)
Both adult and pediatrics	26 (10.5)
Specialty^*^	
Hospital wide	120 (48.6)
More than one specialty	37 (15.0)
Emergency medicine	25 (10.1)
General medicine	21 (8.5)
Intensive Care Unit (ICU)	21 (8.5)
Surgery	19 (7.7)
Obstetrics and Gynecology	3 (1.2)
Oncology	4 (1.6)
Not specified	12 (4.9)
**Disease focus**	
Respiratory tract infections	20 (8.1)
Urinary Tract Infections	16 (6.5)
Bloodstream infections	15 (6.1)
Gastrointestinal infections	10 (4.0)
Trauma and wound infections	5 (2.0)
More than one disease or infection	5 (2.0)
Not disease specific	101 (40.9)
Other diseases or infections^^^	75 (30.4)

* Articles reported multiple responses (percentages add up to >100%).

^ Other diseases and infections included human immunodeficiency virus (HIV), fungal infections, acute exacerbations chronic obstructive pulmonary disease, chorioamnionitis, non-purulent skin and soft tissue infections, and device-associated infections.

ASPs using hospital-wide strategies were reported in nearly half of the articles (48.6%). Emergency departments (10.1%) and intensive care units (8.5%) were the most common specialties where ASPs targeted at the unit level. Additionally, 15% of ASPs were implemented in more than one specialty. Close to half (40.9%) of the studies did not have a specific disease focus. Among strategies that had a specific disease focus, the most common were respiratory tract infections (8.1%), urinary tract infections (6.5%), and bloodstream infections (6.1%).

### Study designs and theories

Table 3 provides specifics about study designs used. Pre- and post- assesment was the most commonly used study designs (90.7%). Single-site designs were used in 69.6% of studies, and multiple-site designs in 29.6% (See Table 3). The mean amount of time spent on preintervention data collection was 14.1 months (SD = 12.4), and the mean amount of time spent on postintervention data collection was 18.3 months (SD = 19.2). A total of 70.0% of articles were not overtly informed by any theory, model, or framework (TMF). Among articles that did report using a TMF, 23% of articles reported using a TMF from the CDC, WHO, Infectious Disease Society of America (IDSA), or other large professional organization.

**Table 3.** Study design characteristics and ASP intervention strategies Table 3 long description.

Characteristic	n (%)
Study designs^*^	
Pre-post design	224 (90.7)
Retrospective design	180 (72.9)
Single-site design	172 (69.6)
Multiple sites design	73 (29.6)
Prospective design	51 (20.6)
Comparison group	31 (12.6)
Clustering	22 (8.9)
Randomization	13 (5.3)
**Study time**	
Mean time in preintervention	14.1 months (SD = 12.4)
Mean time in postintervention	18.3 months (SD = 19.2)
Theories, Models and Frameworks (TMF)^*^	
None used	173 (70.0)
CDC, WHO, IDSA or other large organization TMF	57 (23.1)
Business management or organizational behavior	8 (3.2)
Social science TMF	9 (3.6)
Implementation science TMF	3 (1.2)
Other^^^	5 (2.0)
**ASP team involvement**	
ASP team Led	128 (51.8)
No specific involvement	33 (13.4)
ASP informed and involved	23 (9.3)
Not specified	63 (25.5)
ASP team members^*^	
Medical doctors	109 (44.1)
Pharmacists	112 (45.3)
Microbiology and lab technicians	30 (12.1)
Infection preventionists	19 (7.7)
Registered nurses	26 (10.5)
Informatics and data analysts	8 (3.2)
Not specified	23 (9.3)
**Number of implementation strategies used**	
Multiple strategies (more than one per study)	206 (83.4)
Mean [SD]	2.94 [1.36]
Implementation strategies^*^	
Education or learning	157 (63.6)
Rounding, handshake, audit and feedback, peer comparison	136 (55.1)
New clinical guideline, pathway, policy or decision tree	137 (55.5)
Diagnostics and testing	81 (32.8)
EMR based intervention	66 (26.7)
Prior approval or restrictions	43 (17.4)
Facilitation	34 (13.8)
QI initiatives (PDSA cycle)	26 (10.5)
Other	45 (18.2)
Focus of intervention (Target of Change)^*^	
Clinicians	221 (89.5)
Unit or system	139 (56.3)
Patients	20 (8.1)

* Articles reported multiple responses (total percentages add up to >100%).

^ Other TMF included System Usability Scale (SUS); WAR score for assessing infection in chronic wounds; evidence-based practice guidelines published in American Journal of Health-System Pharmacy; Mendelson‘s nine essential steps for ASP implementation.

### ASP team compositions and involvement

The role and involvement of ASP teams varied across the studies (see Table 3). Fifty one percent of studies were led by an ASP team, although in some studies, ASP teams informed strategies (9.3%) or were not involved at all (13.4%). Pharmacists (45.3%) and medical doctors (44.1%) made up the majority of ASP team members who were involved in the strategy development and delivery.

### Interventions and implementation strategies

Table 3 also describes the strategies used as an effort to improve antimicrobial use. Eighty-three percent (n = 208) of studies used more than one implementation strategy, with an average of three strategies used per study. Strategies targeted at clinicians were used in 89.5% of studies. Such interventions included audit/feedback approaches (eg, handshake stewardship), education, and guidelines at the unit level intended to change individual provider behavior. Interventions targeted at the unit level, including order set changes, automatic stops, and protocols that change workflow (prior approval), were used in 56.3% studies. Finally, interventions that focused on patients (eg, shared decision-making) were only used in 8.1% of studies.

Implementation strategies mostly constituted education, which was used in 63.6% of studies, followed closely by new guidelines, clinical pathways, policies, or decision trees (55.5%) and audit and feedback or handshake stewardship (55.1%). Strategies around diagnosis and testing were used in 32.8% of articles. This strategy involved the appropriate use of laboratory testing to guide patient management, including treatment, to optimize clinical outcomes and limit the spread of antimicrobial resistance. Other strategies included electronic medical records (EMR)-based interventions (26.7%), prior approval or restrictions (17.4%), facilitation (used in 13.8%), and quality improvement initiatives (QI), such as Plan-Do-Study-Act (PDSA) cycles (10.5%).

### Implementation outcomes and effects

Table 4 describes the outcomes of the studies at the level of the implementation strategy, the antimicrobial resource used, and patient outcomes, if any. Forty-three percent of articles did not track any implementation outcome. Among the implementation outcomes that were tracked were fidelity (measuring the degree to which providers’ interventions adhered to guidelines or protocols; 32.0%) and acceptability (24.7%). Of note, 31.6% of articles reported all beneficial effects and 14.8% reported mixed effects (see Table 5).

**Table 4. tbl4:** ASP outcomes tracked Table 4 long description.

Outcomes	n (%)
**Implementation outcomes**	
None	105 (42.5)
Fidelity	79 (32.0)
Acceptability	61 (24.7)
Implementation cost (eg, FTEs)	21 (8.5)
Appropriateness	21 (8.5)
Reach	19 (7.7)
Feasibility	13 (5.3)
Knowledge	11 (4.5)
Sustainment	9 (3.6)
Awareness	7 (2.8)
Adaptation	4 (1.6)
**Resource outcomes**	
Measure of overall antibiotic consumption/amount	152 (61.5)
Drug choice	143 (57.9)
Duration	87 (35.2)
Drug cost	57 (23.1)
New ASP team development and implementation	50 (20.2)
Diagnostic switch	44 (17.8)
Timing of antibiotics	40 (16.2)
Dose of antibiotic	33 (13.4)
IV to oral switch	24 (9.7)
Other	27 (10.9)
**Patient outcomes**	
Length of stay	95 (38.5)
Infections (eg, *Clostridium* Difficile, surgical)	88 (35.6)
Mortality	88 (35.6)
Admissions or readmissions	59 (23.9)
MDRO emergence	44 (17.8)
Allergic or adverse reactions	18 (7.3)
Other	61 (24.7)
None	59 (23.9)

**Table 5.** Reported implementation, patient, and resource effects Table 5 long description.

Reported effects	Implementation outcomes n (%)	Resource utilization outcomes n (%)	Primary patient outcomes n (%)
All beneficial	85 (34.4)	125 (50.6)	54 (21.9)
Mixed	32 (13.0)	106 (42.9)	59 (23.9)
Null	16 (6.5)	15 (6.1)	74 (30.0)
All harmful	3 (1.2)	1 (0.4)	3 (1.2)
None reported	106 (42.9)	—	57 (23.1)

### Resource utilization outcomes and effects [Table tbl4]

Resource utilization outcomes refer to outcomes related to the use of hospital and material resources, such as duration of therapy, associated costs, and technologies involved in the intervention, excluding the implementation strategy itself. Measures of overall antibiotic consumption (eg, days of therapy) were the most tracked (61.5%), followed by change in drug choice in 57.9% of studies (eg, broad to narrow or replacing an inappropriate antibiotic with an appropriate one). Other outcomes that were tracked were duration (eg, average duration of therapy) in 35.2% of studies and cost (antibiotics/drug costs; cost of prescribing and administering antibiotics) in 23.1% of articles. All beneficial effects on resource utilization outcomes were reported in 50.6% of articles, with mixed effects in 42.9% and null effects in 6.1% (see Table 5).

### Resource utilization outcomes by intervention strategy [Table tbl4]

Quality improvement initiatives, such as PDSA cycles, had the highest proportion of all-beneficial resource outcome effects (76.9%), though this strategy was used in a relatively small number of studies (n = 26). Education (52.9%), guideline or pathway implementation (55.5%), and audit and feedback approaches (51.5%) also showed majority beneficial resource effects. Prior approval and restriction strategies were notable in that most studies using this approach reported mixed effects (69.8%), the highest mixed-effects rate of any strategy, suggesting that while these approaches reliably shift antimicrobial use, the nature and direction of that change is variable across contexts. Facilitation had the highest null effects rate among all strategies (11.8%). Across all strategies, all-harmful resource effects were rare, ranging from 0% to 1.5% (see Table 5).

### Primary patient outcomes and effects [Table tbl4]

For primary patient outcomes, that is outcomes related to the health of patients as a result of the healthcare resources applied to them, length of stay (38.5%), infections such as *Clostridioides difficile* and surgical site infection (35.6%), and mortality (35.6%) were the most commonly tracked outcomes. Admissions and readmissions were tracked in 23.9% of studies, and MDRO emergence in 17.8%. Fifty-nine studies (23.6%) did not track any primary patient outcome. Fifty-four (21.6%) studies reported all beneficial effects (ie, either improved patient outcomes or no increase in patient harm due to reduced antibiotic use), 23.9% reported mixed effects, and three studies reported all harmful effects (ie, increased length-of-stay or mortality). Null effects were reported in the remaining 30.0% studies indicating that many efforts neither improved nor harmed patient outcomes (see Table 5).

### Impact of strategies on implementation, resource and patient outcomes

**Figure 2.**
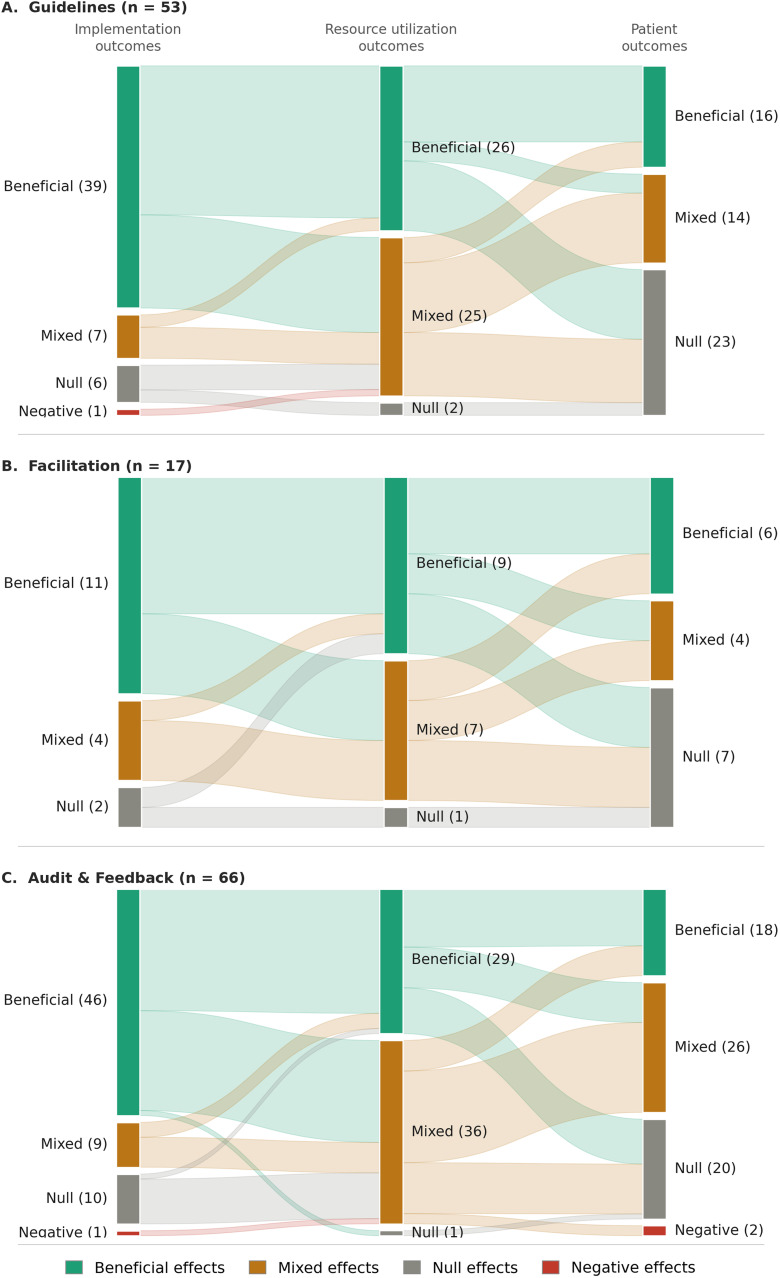
Sankey diagrams of impact of strategies on implementation, resource and patient outcomes.

The Sankey diagrams in Figure 2 further explores the impact of three strategies on implementation, resource, and patient outcomes. Consistently across three strategies, which includes guideline implementation, audit and feedback, and facilitation, beneficial implementation outcomes are most frequently reported. Among studies with beneficial implementation outcomes, the stewardship outcomes tended to be either beneficial or include some beneficial and some null results (ie, mixed). Conversely studies with mixed or null implementation outcomes tended to have mixed or null stewardship and/or patient results. Individual panels also demonstrate outcome variability in the same strategy. Using panel 3 on audit and feedback as an example, while a meaningful proportion of studies with beneficial implementation outcomes also reported beneficial resource and patient-level outcomes, another meaningful proportion reported subsequent mixed outcomes. This demonstrates several possibilities within the literature. First, that audit and feedback works well for some outcomes but not others. Second, that some outcomes may be difficult to measure or not the best indicator of change, like AMR emergence which may require a longer time horizon relative to the length of the study. A third possibility is that different ways of structuring audit and feedback may have different impacts on subsequent outcomes.

## Discussion

This systematic review examined the strategies used by ASPs and their impact on both stewardship-related and implementation outcomes. While this review suggests significant progress in conducting antimicrobial stewardship and monitoring its value, our results also suggest areas where stewardship strategies and evaluation efforts could be improved. For instance, most studies used pre and post comparative analyses as well as single site research designs. More rigorous research designs including multiple sites, randomization of strategies, and measuring consistent outcomes across sites would be highly beneficial. Our findings align with prior research demonstrating that ASPs are predominantly implemented in inpatient settings, particularly academic and teaching hospitals in high resource countries.^16,17^ Given that a substantial portion of antibiotic misuse occurs in outpatient and global settings, where nearly 30% of prescriptions are deemed unnecessary,^18,19^ this gap represents a critical missed opportunity.^20^

Our findings also support ways the field of implementation science could support ASP activities and research in alignment with more recent calls to focus more heavily on designing stewardship strategies that consider behavior change.^21–23^ Although ASP strategies included in this review aimed to influence prescriber behavior, they rarely incorporated theoretical frameworks that explicitly address how behavior change occurs.^10,24^ Prior research suggests that behavioral change interventions rooted in established implementation science models, such as the Theory of Planned Behavior,^25^ or the Consolidated Framework for Implementation Research (CFIR),^26^ are more likely to achieve sustained improvements in prescribing behavior. Furthermore, there are many existing tools and resources to help clinicians and researchers use models to guide their behavior change efforts.^27–29^

One of the fundamental problems stimulating implementation science is the inability to determine whether an intervention is not beneficial to recipients or if it simply was not implemented well.^13,30^ Our review suggests a parallel where it is difficult to discern whether the ASP strategy, for instance an audit and feedback strategy, either does not have the intended influence on clinician behavior or if it is simply not implemented in the most effective way. In addition to frameworks to guide behavior change strategies, a wealth of implementation science evidence is available to inform ASP strategy design in similar contexts, especially with common strategies like audit and feedback. In addition, without examining the implementation outcomes—like whether a clinician reviews their feedback—it is difficult to discern why the strategy is ineffective or if parts of the strategy are effective, but others are not in the instance of using multiple strategies together. This aligns with previous findings that ASP systematic reviews often focus on clinical outcomes such as AMR rates while lacking implementation factors that determine an intervention’s success or failure.^31,32^ We encourage ASPs to track these indicators to provide a better understanding of whether an ASP strategy is working as intended or if modifications to the strategy are necessary.

### Limitations

While this review provides valuable insights into ASP implementation, there are several limitations to consider. First, only articles that were published and available in the English language were included. Second, the inclusion criteria may have excluded relevant studies that focused on informal or unpublished ASP efforts. However, given the large number of articles reviewed, we feel that the results from the review are representative of the literature at the time. The heterogeneity in reporting of implementation outcomes further complicates the synthesis of findings and highlights the need for greater standardization in ASP research. Lastly, data were extracted using definitions developed by the researchers and based only on the material reported. For example, investigators may have implicitly used a guiding framework without reporting it in the article.

### Conclusions

While ASPs have proven effective in optimizing antimicrobial use, their full potential has yet to be realized. Expanding ASPs to outpatient and community-based settings will help address antimicrobial misuse in a broader range of clinical environments. By incorporating insights from implementation science, ASPs can be designed and evaluated more systematically, ensuring that they achieve lasting improvements in antimicrobial stewardship. Addressing these gaps will be critical for enhancing the long-term success of ASPs and mitigating the growing threat of antimicrobial resistance.

## Supporting information

10.1017/ash.2026.10769.sm001 McKay et al. supplementary material 1 McKay et al. supplementary material

10.1017/ash.2026.10769.sm002 McKay et al. supplementary material 2 McKay et al. supplementary material

10.1017/ash.2026.10769.sm003 McKay et al. supplementary material 3 McKay et al. supplementary material

## Data Availability

Data are available from the first author upon reasonable request.

## Supplementary material

The supplementary material for this article can be found at https://doi.org/10.1017/ash.2026.10769.

## Table 1 Long description

A table categorizing and defining various outcome types related to implementation and resource utilization. The table is divided into three main categories: Implementation outcomes, Resource utilization outcomes, and Primary patient outcomes. Each category contains several outcome types with their respective definitions. Implementation outcomes include Knowledge, Awareness, Acceptability, Feasibility, Appropriateness, Fidelity, Reach, Adaptation, Sustainment, and Implementation Cost. Resource utilization outcomes include Overall consumption, Drug choice, Duration, Dose, IV-to-oral switch, Timing, Diagnostic switch, and Resource Cost. Primary patient outcomes include Mortality, Length of stay, Infections, Admissions/readmissions, and MDRO emergence. The table also includes an outcome effect classification with categories such as All beneficial, All harmful, Mixed, and Null. Each row in the table lists an outcome type followed by its definition. For example, under Implementation outcomes, Knowledge is defined as the extent to which providers understand the intervention or prescribing practice being implemented. Under Resource utilization outcomes, Overall consumption is defined as the total antibiotic use at the unit or institutional level. Under Primary patient outcomes, Mortality is defined as all-cause or infection-related death during the study period.

Navigate back to Table 1.

## Table 2 Long description

A table detailing the characteristics of included studies, focusing on regions, settings, age of patient population, specialty, and disease focus. The table has 20 rows and 6 columns. Column headers are Characteristic, Region, Settings, Age of patient population, Specialty, and Disease focus. Row labels include various categories under each characteristic. Values are presented as counts and percentages. Notable trends include the majority of studies conducted in the USA (51.6%), inpatient settings (80.2%), academic or teaching adult hospitals (30.0%), and adult patient populations (75.3%). Specialties are broadly distributed with hospital-wide being the most common (48.6%), and disease focus is varied with a significant portion not disease-specific (40.9%).

Navigate back to Table 2.

## Table 3 Long description

A table detailing study design characteristics and ASP intervention strategies. The table has 12 rows and 2 columns. The columns are labeled ‘Characteristic’ and ‘n (%)’. The rows are labeled with various characteristics and their corresponding values. Row 1: Study designs, Pre-post design, 224 (90.7 percent). Row 2: Study designs, Retrospective design, 180 (72.9 percent). Row 3: Study designs, Single-site design, 172 (69.6 percent). Row 4: Study designs, Multiple sites design, 73 (29.6 percent). Row 5: Study designs, Prospective design, 51 (20.6 percent). Row 6: Study designs, Comparison group, 31 (12.6 percent). Row 7: Study designs, Clustering, 22 (8.9 percent). Row 8: Study designs, Randomization, 13 (5.3 percent). Row 9: Study time, Mean time in preintervention, 14.1 months (SD = 12.4). Row 10: Study time, Mean time in postintervention, 18.3 months (SD = 19.2). Row 11: Theories, Models and Frameworks (TMF), None used, 173 (70.0 percent). Row 12: Theories, Models and Frameworks (TMF), CDC, WHO, IDSA or other large organization TMF, 57 (23.1 percent).

Navigate back to Table 3.

## Table 4 Long description

A table categorizing outcomes tracked in studies related to antimicrobial stewardship programs. The table has three main categories: Implementation outcomes, Resource outcomes, and Patient outcomes. Each category lists specific outcomes with corresponding counts and percentages. Implementation outcomes include categories such as Fidelity, Acceptability, Implementation cost, Appropriateness, Reach, Feasibility, Knowledge, Sustainment, Awareness, and Adaptation. Resource outcomes include Measure of overall antibiotic consumption/amount, Drug choice, Duration, Drug cost, New ASP team development and implementation, Diagnostic switch, Timing of antibiotics, Dose of antibiotic, IV to oral switch, and Other. Patient outcomes include Length of stay, Infections, Mortality, Admissions or readmissions, MDRO emergence, Allergic or adverse reactions, and Other. The table lists the number and percentage of studies tracking each outcome. For example, 105 studies (42.5 percent) did not track any implementation outcome, while 79 studies (32.0 percent) tracked Fidelity. In the Resource outcomes category, 152 studies (61.5 percent) tracked the Measure of overall antibiotic consumption/amount, and 143 studies (57.9 percent) tracked Drug choice. In the Patient outcomes category, 95 studies (38.5 percent) tracked Length of stay, and 88 studies (35.6 percent) tracked both Infections and Mortality.

Navigate back to Table 4.

## Table 5 Long description

A table comparing the outcomes of studies at the level of the implementation strategy, the antimicrobial resource used, and patient outcomes. The table has five rows and four columns. The columns are labeled Reported effects, Implementation outcomes n (%), Resource utilization outcomes n (%), and Primary patient outcomes n (%). The rows are labeled All beneficial, Mixed, Null, All harmful, and None reported. Row 1: All beneficial, 85 (34.4), 125 (50.6), 54 (21.9). Row 2: Mixed, 32 (13.0), 106 (42.9), 59 (23.9). Row 3: Null, 16 (6.5), 15 (6.1), 74 (30.0). Row 4: All harmful, 3 (1.2), 1 (0.4), 3 (1.2). Row 5: None reported, 106 (42.9), — , 57 (23.1).

Navigate back to Table 5.
