# Supplementary material for: Systematic review of implementing stewardship activities to promote appropriate antimicrobial use
Source: Antimicrob Steward Healthc Epidemiol. 2026 Aug 3;6(1):e233. doi: 10.1017/ash.2026.10769 (PMC13435156; doi:10.1017/ash.2026.10769)
Supplement: Supplementary file 1 [file S2732494X26107694sup002.pdf]

Form 1

Paper ID - What is the article ID? ______________________

Extractor Name:
- ◯ Kelly
- ◯ Ginger
- ◯ Sara
- ◯ Collin
- ◯ Luke
- ◯ Josh
- ◯ Matt
- ◯ Lauren
- ◯ Harry
- ◯ McKenzie
- ◯ Tyler
- ◯ Emmanuel

Who is the author? ______________________

What is the publication year?
- ◯ 1995
- ◯ 1996
- ◯ 1997
- ◯ 1998
- ◯ 1999
- ◯ 2000
- ◯ 2001
- ◯ 2002
- ◯ 2003
- ◯ 2004
- ◯ 2005
- ◯ 2006
- ◯ 2007
- ◯ 2008
- ◯ 2009
- ◯ 2010
- ◯ 2011
- ◯ 2012
- ◯ 2013
- ◯ 2014
- ◯ 2015
- ◯ 2016
- ◯ 2017
- ◯ 2018
- ◯ 2019
- ◯ 2020
- ◯ 2021
- ◯ 2022
- ◯ Other

Other: Enter the publication year here. ______________________

What is the article title? ______________________

Journal Name ______________________

What is the amount of time spent in pre-intervention data collection? (list in months)	__________________________________
What is the amount of time that post-intervention data was collected? (list in months)<br><br>If just the month and year is listed, count that as a whole month.	__________________________________
In what country was the study conducted?	◯ USA<br>◯ Australia<br>◯ Brazil<br>◯ Canada<br>◯ China<br>◯ France<br>◯ Germany<br>◯ Greece<br>◯ India<br>◯ Italy<br>◯ Japan<br>◯ Malaysia<br>◯ Norway<br>◯ Portugal<br>◯ Qatar<br>◯ Saudi Arabia<br>◯ Spain<br>◯ Taiwan<br>◯ UK<br>◯ United Arab Emirates<br>◯ Vietnam<br>◯ Multinational<br>◯ Other
If the country selected was 'other', please list the country here:	__________________________________
If the country selected was 'multinational', please enter countries here separated by a comma:	__________________________________
Please select the rurality of the study setting:<br><br>Urban is defined as population of 50,000 or more.<br><br>Please avoid searching online and make determination based on what's described in the paper.	◯ Urban setting<br>◯ Rural setting<br>◯ Both urban and rural<br>◯ Not specified/Not reported
In what healthcare setting did this take place?<br><br>If it is a multi-site study, and all the sites are the same type, select inpatient or outpatient and then indicate the type. Select multiple settings if it includes a combination of types (an entire system of hospitals is likely more than 2)<br><br>Please avoid searching online and make determination based on what's described in the paper.	◯ Inpatient<br>◯ Outpatient<br>◯ Multiple Settings [any two or more different options above]<br>◯ Not specified/not reported

Please select the type of inpatient setting:	◯ Academic/teaching adult hospital [this can be a tertiary hospital, but have residents or are affiliated with medial school]<br>◯ Academic/teaching children's hospital [this can be a tertiary hospital, but have residents or are affiliated with medial school]<br>◯ Critical access hospital<br>◯ Veteran's hospital<br>◯ Community hospital [including tertiary care]<br>◯ Long-term care/Nursing home<br>◯ Other
You selected "other" inpatient setting. Please specify:	__________________________________
Please select the type of outpatient setting:	◯ Urgent care<br>◯ Primary care practice<br>◯ Other
You selected "other" outpatient setting. Please specify:	__________________________________
If the study took place in multiple settings, please list them here:	__________________________________
What was the medical specialty that was involved?	☐ This was targeted hospital wide<br>☐ Surgery<br>☐ OB/GYN<br>☐ Oncology<br>☐ ICU<br>☐ Emergency Medicine<br>☐ General Medicine/Hospitalist<br>☐ Other<br>☐ Not described/not specified<br>☐ Not applicable
You selected that this was an 'other' medical specialty. Please describe here:	__________________________________
What was the disease/diagnosis targeted?<br><br>Here, list the disease or specific medical diagnosis the ASP intervention was targeting (i.e. sepsis).<br><br>if the whole hospital was targeted, or there was not one specific disease, list this is as 'not disease specific'	__________________________________
What is the patient population included?<br><br>(If the population age is not described, please select "adult.")	◯ Adult (only 18+)<br>◯ Pediatric (only patients under 18)<br>◯ Adult and pediatrics<br>◯ Not applicable
How was the size of the organization described?	☐ number of staff<br>☐ number of patients/beds<br>☐ Other<br>☐ Not described/not specified<br>☐ Not applicable

You selected "other" organization description. Please specify:	__________________________________
What is the number of patients/beds? if multiple hospitals, add together	__________________________________
What is the number of staff? if multiple hospitals, add together	__________________________________
Did the study design include any of these?	☐ Randomization - participants in the study (individuals or organizations) are randomly assigned to intervention groups<br>☐ clustering - individuals or organizations are grouped in some way in the study<br>☐ Multiple Sites - There is more than one organization or site involved in the study<br>☐ Single Site - this only has one unit of organizational measurement (hospital, clinic, etc.)<br>☐ Pre/Post design - measures the outcome of interest both before and after a particular intervention is delivered<br>☐ Comparison group - a group with no intervention, represents a control group and way to understand the difference in two or more groups<br>☐ Retrospective - the data was collected looking back, the study was designed and data was collected after the time period (looking back)<br>☐ Prospective design - ** to include this, the paper must specifically say it was prospective** individuals are followed over time, and data about them is collected as their characteristics or circumstances change
How many sites are involved in the study?	__________________________________
copy and paste the stated design	____
Was the sample measured by the patient number or clinician number?<br><br>If the sample is something like "days of therapy/1000 patient days", list as other and then explain.	☐ Patient<br>☐ Clinician<br>☐ Number of antibiotic prescriptions [not associated with patient number]<br>☐ Other<br>☐ Not described/not specified<br>☐ Not applicable
You selected "other" sample measurement. Please specify:	__________________________________
What was the overall patient sample size? if multiple hospitals, add together	__________________________________
What was the overall clinician sample size? if multiple hospitals, add together	__________________________________
What was the overall number of antibiotic prescriptions?	__________________________________

What were the ASP interventions used?<br><br>(Papers needed to have at least one to meet inclusion criteria)<br><br>check here even if used at some, but not all, of the sites in the study	☐ EMR based intervention (integrations such as order sets, prompts, etc. -- this is not running reports for audits)<br>☐ Rounding/hanshake stewardship/audit and feedback/peer comparison<br>☐ QI initiatives (PDSA cycle)<br>☐ diagnostics/testing (both the introduction and the limiting) -- this is conducting a new test, or diagnosis improvement to then have more precise antibiotics (refers to the appropriate use of laboratory testing to guide patient management, including treatment, in order to optimize clinical outcomes and limit the spread of antimicrobial resistance)<br>☐ Education/learning<br>☐ prior approval/restrictions<br>☐ new guideline/clinical pathway/policy/decision tree<br>☐ Facilitation (looking for when a team member is conceptualized as a facilitator, but might be person, or team of people, that are intentionally identified as individuals who help build capacity and facilitate the change process. Sometimes described as a "champion," but verify that the "champion" is engaging in facilitation work)<br>☐ Other
You selected "other" ASP intervention. Please list:	__________________________________
Copy and paste the ASP implementation strategy (strategies) that were described. This includes use at any site.<br><br>Paper on implementation strategies: https://implementationscience.biomedcentral.com/articles/10.1186/s13012-015-0209-1	__________________________________
Describe the use of theory in the paper (these need to have been specifically identified or cited in the paper).<br><br>Will likely be found in the "methods" section and will inform the study design.	☐ Implementation Science theory/framework/model<br>☐ social science theory/framework/model<br>☐ business management/organizational behavior<br>☐ CDC/WHO/IDSA other large health organization framework<br>☐ Other<br>☐ None used
You selected "other" theory. Please list:	__________________________________
What was the level of ASP team involvement in the intervention (design or delivery)?<br><br>(We are not concerned about who is involved in the research team or the study design.)	◯ No specific ASP team involvement<br>◯ ASP team informed and involved (MD or Pharmacist)<br>◯ ASP team led/driven (MD or pharmacist)<br>◯ This paper doesn't specifically address this // it is unclear who is involved in the strategy because nothing about the strategy team is described

Who were the ASP team members involved in the strategy?<br><br>(which team member/members delivered the intervention?)	☐ MD<br>☐ RN<br>☐ Pharmacist<br>☐ Infection preventionists<br>☐ microbiology/lab<br>☐ IT/Informatics/Data analyst<br>☐ Other<br>☐ ASP team was involved in development but another team conducted/delivered the intervention<br>☐ This was not specifically addressed in the paper
You selected "other" ASP team member involved. Please specify.	__________________________________
What was the focus of the intervention (or the target for change)?	☐ Patient [education, shared decision making]<br>☐ Clinicians [audit/feedback, education, handshake stewardship, guidelines that are at the unit level but intended to change individual provider behavior]<br>☐ Unit/System [order set changes, automatic stops, protocols that change workflow (prior approval), interventions that are meant to change how the system functions]<br>☐ Other<br>☐ Not described/not specified<br>☐ Not applicable
You selected "other" intervention focus. Please specify:	__________________________________
What were the implementation science outcomes in the study?<br><br>https://www.ncbi.nlm.nih.gov/pmc/articles/PMC3068522/table/Tab1/?report=objectonly<br><br>*we think it's unlikely there will be many of these in the papers, and it will be directly addressed in the paper*<br><br>General reminder: this is about the intervention and is usually measured at the provider level; this is not about the drug appropriateness or acceptability/// this is focused on the diagnostic or prescribing practices<br><br>This is about the extent to which an intervention is adopted into practice, a strategy influences perceptions of, actual adoption, or better use of an intervention (i.e., resource outcome).	☐ Knowledge (knowledge about the intervention/practice that is being implemented)<br>☐ Awareness (awareness of the protocol change, guideline, new diagnostic practice)<br>☐ Acceptability (this is the provider perception of acceptability, i.e. we tested the acceptability of the intervention in the setting. Provider accepts/follows ASP recommendations)<br>☐ Feasibility (how providers think the intervention is feasible; example the feasibility of changing the prescribing or diagnostic practices)<br>☐ Appropriateness (provider perception of compatibility; Perceived fit; relevance; compatibility; suitability; usefulness; practicability; more of a qualitative measure)<br>☐ Cost (this is the implementation cost, something like the cost of half of a pharmacists time or the education or strategy cost)<br>☐ Fidelity (measuring the degree to which providers adhere to guidelines or protocol)<br>☐ Reach (penetration into the setting; Level of institutionalization, Spread, Service access)<br>☐ Adaptation (changes to the intervention to facilitate implementation into unique contexts)<br>☐ Sustainment (measures of continuation of the intervention over time)<br>☐ None

What were the resource utilization study outcomes?

(Note: papers needed to have at least one to meet inclusion criteria.)

This is about hospital/material use (the amount of something consumed). This focuses on things like the overall use of beds, drugs (antibiotics), and costs associated with this. This can also include the use of technologies associated with the intervention or staff effort that goes into the program/intervention but not the strategy.

- ☐ New ASP team development and implementation (initiation of a new ASP program, not an intervention within the program... the ASP team is formed and is still in place, the ASP team has 2 pharmacists, etc.; some reported outcome regarding the team)
- ☐ Duration (e.g., average duration of therapy; looking at how long the drug is prescribed for a disease -- often reducing; reduction in length of antibiotics)
- ☐ Drug Choice (e.g., broad to narrow, beta lactam positive?) (something about changing the drug that is selected to be more appropriate --- this is the appropriateness of antibiotics, like did they choose the right antibiotic for the situation -- as compared to appropriateness as described above)
- ☐ Timing of antibiotic (this could be the frequency of delivery or the time from diagnosis to treatment -- example is the ER studies where they want to increase the antibiotic delivery speed)
- ☐ IV to oral switch (number or percent of IV drugs that were switched to be given orally, number of people converted to oral antibiotics)
- ☐ Diagnostic switch: Ordered when indicated (reducing inappropriate diagnostics or increasing precision diagnostics to ensure more appropriate antibiotic choice -- but this outcome focuses on the diagnostics)
- ☐ Cost (antibiotics/drug costs; cost of prescribing and giving antibiotics)
- ☐ Measure of overall antibiotic consumption/amount (often measured at the unit or hospital level as opposed to the individual level; overall consumption amount and/or rate, # of patients prescribed, sometimes calculated as percent/rate/aggregate, defined daily dose, days of therapy)
- ☐ Dose of antibiotic (measure of the amount given at one time, with the idea that it has the intended therapeutic effect NOT the total number of doses, which would be duration)
- ☐ Other
- ☐ None

What were the other resource utilization outcomes?

____

What was the primary patient outcomes?

These are outcomes about the health of patients as a result of the healthcare resources applied to them. These outcomes may not be tied directly to specific patients, but also may be a global outcome i.e., emergence of a resistant strain of bacteria in a hospital.

- ☐ Infection due to antimicrobial practice change (e.g., c. diff, SSI, or none)
- ☐ MDRO emergence
- ☐ Mortality
- ☐ Admissions/Readmissions
- ☐ Length of Stay
- ☐ Other
- ☐ None

You selected "other" patient outcome. Please list:

____

Please copy/paste the results section of the abstract here:

____

What were the reported effects of the implementation science outcomes of this study?

(record what is reported in the results section.)

Note: by "positive effects" we mean beneficial improvements like reduced mortality or length-of-stay; by negative we mean harmful effects like increased mortality or lengthier stays. As opposed to positive and negative direction. If a paper mentions that no discernible changes in outcomes were observed, then enter "null." We are actually hoping that this would be the case in a lot of situations. Mixed would be observing both improvement and harmful outcomes (or positive/no change or negative/no change)

- ◯ All positive effects
- ◯ All negative effects
- ◯ Mixed effects reported
- ◯ Null
- ◯ No reported effects

Please enter the sentence(s) with negative, mixed, or null results.

____

What were the reported effects for the resource utilization outcomes of this study?

(Record what is reported in the results section.)

Note: by "positive effects" we mean beneficial improvements like reduced mortality or length-of-stay; by negative we mean harmful effects like increased mortality or lengthier stays. As opposed to positive and negative direction. If a paper mentions that no discernible changes in outcomes were observed, then enter "null." We are actually hoping that this would be the case in a lot of situations. Mixed would be observing both improvement and harmful outcomes (or positive/no change or negative/no change)

- ◯ All positive effects
- ◯ All negative effects
- ◯ Mixed effects reported
- ◯ Null

Please enter the sentence(s) with negative, mixed or null results.

____

What were the reported effects on the patient (clinical) outcomes of this study?

(Record what is reported in the results section.)

Note: by "positive effects" we mean beneficial improvements like reduced mortality or length-of-stay; by negative we mean harmful effects like increased mortality or lengthier stays. As opposed to positive and negative direction. If a paper mentions that no discernible changes in outcomes were observed, then enter "null." We are actually hoping that this would be the case in a lot of situations. Mixed would be observing both improvement and harmful outcomes (or positive/no change or negative/no change)

- ◯ All positive effects
- ◯ All negative effects
- ◯ Mixed effects reported
- ◯ Null
- ◯ No reported effects

Please enter the sentence(s) with negative, mixed, or null results.

____

Does this paper meet inclusion criteria?<br><br>Common reasons to exclude are: no comparison group (e.g. control group or pre-implementation data/group), no ASP implementation strategies in paper, no resource utilization outcomes, no formal statistical analysis.<br><br>For full inclusion/exclusion criteria see this document in box: Operatic Systematic Review Exclusion and InclusionV2.docx	◯ yes<br>◯ no--exclude
Please note why this paper is being excluded:	__________________________________
