# Supplementary material for: Systematic review of implementing stewardship activities to promote appropriate antimicrobial use
Source: Antimicrob Steward Healthc Epidemiol. 2026 Aug 3;6(1):e233. doi: 10.1017/ash.2026.10769 (PMC13435156; doi:10.1017/ash.2026.10769)
Supplement: Supplementary file 2 [file S2732494X26107694sup001.docx]

**PRISMA 2020 Checklist**

Study Title: Systematic Review of Implementing Stewardship Activities to Promote Appropriate Antimicrobial Use

| **Section** | **Topic** | **#** | **Checklist item** | **Location in manuscript** |
| --- | --- | --- | --- | --- |
| ***Title*** | | | | |
| Title | Title | 1 | Identify the report as a systematic review. | Title page |
| ***Abstract*** | | | | |
| Abstract | Abstract | 2 | See the PRISMA 2020 for Abstracts checklist. | Abstract, p. 1 |
| ***Introduction*** | | | | |
| Introduction | Rationale | 3 | Describe the rationale for the review in the context of existing knowledge. | Introduction, pp. 3–4 |
|  | Objectives | 4 | Provide an explicit statement of the objective(s) or question(s) the review addresses. | Introduction, p. 4 |
| ***Methods*** | | | | |
| Methods | Eligibility criteria | 5 | Specify the inclusion and exclusion criteria for the review and how studies were grouped for the syntheses. | Methods, Inclusion/Exclusion Criteria, p. 5 |
|  | Information sources | 6 | Specify all databases, registers, websites, organisations, reference lists and other sources searched or consulted to identify studies. Specify the date when each source was last searched or consulted. | Methods, Search Methodology, p. 5: Embase, Ovid MEDLINE, Scopus, CDSR, CENTRAL, CINAHL Plus, ASHE, ClinicalTrials.gov. Searched October 29, 2021; updated February 26, 2025. |
|  | Search strategy | 7 | Present the full search strategies for all databases, registers and websites, including any filters and limits used. | Supplemental File — Search Strategies Appendix |
|  | Selection process | 8 | Specify the methods used to decide whether a study met the inclusion criteria of the review, including how many reviewers screened each record and whether they worked independently. | Methods, p. 5: two independent reviewers; conflicts resolved by discussion or third reviewer |
|  | Data collection process | 9 | Specify the methods used to collect data from reports, including how many reviewers collected data from each report and whether they worked independently. | Methods, Extraction, p. 5: two independent reviewers; discrepancies discussed and resolved |
|  | Data items — outcomes | 10a | List and define all outcomes for which data were sought. Specify whether all results compatible with each outcome domain in each study were sought. | Methods, Extraction, p. 5; Supplemental File 1 (variable codebook) |
|  | Data items — other variables | 10b | List and define all other variables for which data were sought. Describe any assumptions made about missing or unclear information. | Supplemental File 1: article identifiers, research context, study design, ASP strategies |
|  | Study risk of bias assessment | 11 | Specify the methods used to assess risk of bias in the included studies, including details of the tool(s) used. | Not formally assessed with a standardized tool. Discussed in limitations; recommended for future reviews. |
|  | Effect measures | 12 | Specify for each outcome the effect measure(s) used in the synthesis or presentation of results. | Methods, Data Management, p. 6: descriptive statistics; effects classified as beneficial, mixed, null, or harmful based on direction reported by authors |
|  | Synthesis methods | 13a | Describe the processes used to decide which studies were eligible for each synthesis. | All 247 included studies contributed to descriptive synthesis. No meta-analysis performed. |
|  |  | 13b | Describe any methods required to prepare the data for presentation or synthesis. | Methods, p. 6: data managed in R; reviewed for missingness; variable categories collapsed as appropriate |
|  |  | 13c | Describe any methods used to tabulate or visually display results of individual studies and syntheses. | Tables 1–3; Figures 1–3; Sankey diagrams (Figure X) |
|  |  | 13d | Describe any methods used to synthesize results and provide a rationale for the choice(s). If meta-analysis was performed, describe the model(s) used. | Descriptive synthesis only; heterogeneity in study designs precluded meta-analysis. Rationale noted in Limitations. |
|  |  | 13e | Describe any methods used to explore possible causes of heterogeneity among study results. | Not formally explored; variability noted descriptively by region, setting, and strategy type |
|  |  | 13f | Describe any sensitivity analyses conducted to assess robustness of the synthesized results. | No sensitivity analyses conducted |
|  | Reporting bias assessment | 14 | Describe any methods used to assess risk of bias due to missing results in a synthesis. | Not formally assessed; acknowledged as limitation |
|  | Certainty assessment | 15 | Describe any methods used to assess certainty (or confidence) in the body of evidence for an outcome. | Not assessed; noted as limitation given variability in study quality |
| ***Results*** | | | | |
| Results | Study selection | 16a | Describe the results of the search and selection process, ideally using a flow diagram. | Results, p. 6; Figure 1 (PRISMA flow diagram): 2,035 records identified; 17 duplicates removed; 1,253 excluded at abstract screening; 515 excluded at full-text; 247 included |
|  | Excluded studies | 16b | Cite studies that might appear to meet the inclusion criteria but which were excluded, and explain why. | Exclusion reasons reported in aggregate in Figure 1. Individual excluded study citations not provided. |
|  | Study characteristics | 17 | Cite each included study and present its characteristics. | Results, Table 1; full study list in supplemental materials |
|  | Risk of bias in studies | 18 | Present assessments of risk of bias for each included study. | Not formally assessed at individual study level; discussed in aggregate in Discussion |
|  | Results of individual studies | 19 | For all outcomes, present summary statistics for each study and effect estimates where appropriate. | Descriptive review; results summarized across studies in Tables 2–3 and Figures 2–3 |
|  | Results of syntheses | 20a | For each synthesis, briefly summarise the characteristics and risk of bias among contributing studies. | Tables 2–3 summarize study design characteristics and outcomes by strategy type |
|  |  | 20b | Present results of all statistical syntheses conducted. | No meta-analysis performed; descriptive frequencies and percentages reported |
|  |  | 20c | Present results of all investigations of possible causes of heterogeneity among study results. | Not applicable |
|  |  | 20d | Present results of all sensitivity analyses conducted. | Not applicable |
|  | Reporting biases | 21 | Present assessments of risk of bias due to missing results for each synthesis assessed. | Not assessed; acknowledged as limitation |
|  | Certainty of evidence | 22 | Present assessments of certainty in the body of evidence for each outcome assessed. | Not formally assessed; variability in study quality noted in Limitations |
| ***Discussion*** | | | | |
| Discussion | Interpretation | 23a | Provide a general interpretation of the results in the context of other evidence. | Discussion, pp. 7–10 |
|  | Limitations of evidence | 23b | Discuss any limitations of the evidence included in the review. | Discussion, Limitations, p. 10 |
|  | Limitations of review | 23c | Discuss any limitations of the review processes used. | Discussion, Limitations, p. 10 |
|  | Implications | 23d | Discuss implications of the results for practice, policy, and future research. | Discussion, Future Directions, p. 10 |
| ***Other Information*** | | | | |
| Other information | Registration | 24a | Provide registration information for the review, including register name and registration number, or state that the review was not registered. | The review was not registered |
|  | Protocol access | 24b | Indicate where the review protocol can be accessed, or state that a protocol was not prepared. | Study protocol was not published online |
|  | Protocol amendments | 24c | Describe and explain any amendments to information provided at registration or in the protocol. | n/a |
|  | Support | 25 | Describe sources of financial or non-financial support for the review, and the role of the funders. | Financial Support section: R01HS026742 (Newland) and R18HS030070 (Newland & McKay), AHRQ |
|  | Competing interests | 26 | Declare any competing interests of review authors. | Conflicts of Interest: authors declare no conflicts of interest |
|  | Data availability | 27 | Report which of the following are publicly available: data collection forms; extracted data; analytic code; other materials. | Data Availability: data available from first author on request; Supplemental File 1 included; search strategies in supplemental appendix. |
|  | | | | |
