# Supplementary material for: Systematic review of implementing stewardship activities to promote appropriate antimicrobial use
Source: Antimicrob Steward Healthc Epidemiol. 2026 Aug 3;6(1):e233. doi: 10.1017/ash.2026.10769 (PMC13435156; doi:10.1017/ash.2026.10769)
Supplement: Supplementary file 3 [file S2732494X26107694sup003.docx]

| **Supplemental File 1: Variables extracted and their descriptions** | |
| --- | --- |
| **Variable** | **Description** |
| **Time spent in Pre- or Post-Intervention** | The number of months variables were monitored and analyzed before or after the intervention took place or began. This was sometimes clearly defined in methods. If results or figures measured a variable for longer than the described intervention period, the larger interval was recorded (e.g. the preintervention period was defined as 12 months but a figure showed retrospective data on antibiotic consumption for three years prior to the intervention so we recorded 36 months). If a study described a pre-intervention, intervention, and subsequent post-intervention period, anything before the intervention was recorded as pre and the periods in which the intervention took place and beyond were combined in post. RCTs generally had 0 months pre. Intervals of less than a month were expressed as a decimal. If just the month and year was listed, the interval was described as 1 month. |
| **Study Setting** | *Rural*: Study settings were determined to be rural if the authors expressly described the geographic area as having a population of <50,000.  *Urban*: Study settings were determined to be urban if the authors expressly described it as such, or if the authors described the setting as having a population of >= 50,000.  *Both urban and rural*: The intervention took place in more than one of the above settings, usually in multi-site studies or studies that took place across a healthcare system. |
| **Medical Specialty Involved** | This captured the medical specialty that was the target of the intervention, or whether an intervention targeted the entire hospital or healthcare setting. |
| **Disease or Diagnosis Targeted** | This captured which disease treatment the ASP intervention was targeting (e.g. sepsis, urinary tract infection, etc..). Interventions that were not related to specific disease or targeting the whole healthcare setting were described as "not disease specific." |
| **Patient Population** | *Adult*: The intervention only impacted patients 18+. Studies that did not describe the age of their patient population were assumed to be working with adult patients.  *Pediatric*: The intervention only impacted patients under 18.  *Adult and pediatric*: The study population was described as including both adult and pediatric patients. |
| **Size of Organization** | For multi-site studies, totals reflect the aggregate number of patients/beds or staff across all sites. |
| **Study Design** | *Randomization*: Selected when participants in the study, either individuals or organizations, are randomly assigned to intervention groups  *Multiple sites*: Selected when more than one organization or site was involved in the study.  *Single site*: Selected when the study involves only one unit of organizational measurement, such as a hospital, clinic, etc.  *Pre-post design*: Selected when a study measured the outcome of interest both before and after a particular intervention is delivered.  *Comparison-group*: Selected when a study involved a group with no intervention/control group.  *Retrospective*: Selected when study data was not collected in real time but looking back at a certain period of time.  *Prospective*: Selected when data about individuals or organizations were collected in real time as their characteristics or circumstances change. Randomized control trials were assumed to be prospective if data collection approach was not described in the paper. |
| **Sample Measurement** | Captured what was being measured in the sample. Most papers presented data related to patients, clinicians, or number of antibiotic prescriptions (not associated with the number of patients). Some papers lacked a sample and instead described prescription rates. For this reason, we captured rates such as "days of therapy/1000 patient days" as "other" when they appeared. For multi-site studies, totals reflect the aggregate number of patients, clinicians, or antibiotic prescriptions across all sites. |
| **ASP Interventions** | For multi-site studies, interventions needed to be in place in at least one site to be included.  *EMR-based interventions*:  Integrations such as order sets or prompts.  *Rounding and handshake stewardship*: Face-to-face, interpersonal stewardship activities that included relationship building, audit and feedback, or peer comparison.  *QI initiatives*: Quality improvement initiatives occurring within the institution meant to impact antimicrobial use. Included PDSA cycles.  *Diagnostics and testing interventions*: Diagnostic improvements, through the introduction or limiting of tests, intended to deliver more precise antibiotics.  *Education/learning*: Initiatives meant to teach individuals about better stewardship practices. Workshops, learning modules, posters, and newsletters are all examples. Target examples included patients, clinicians, or administrators.  *Prior approval or restrictions*: Institutionalized policies that limited the prescribing of specific or classes of antimicrobials or made it easier to prescribe preferred antimicrobials.  *New guidelines*: Institutionalized policies, pathways, or decision trees that guided clinicians as they made prescribing choices.  *Facilitation*: when an individual or a team of people engaged with others in the healthcare setting to build capacity for the effective delivery of stewardship interventions. |
| **Use of Theory** | Theories needed to have been specifically cited or identified in the papers. This included when interventions were designed to reflect frameworks or guidelines from large healthcare organizations such as CDC or WHO. |
| **Level of ASP Involvement** | *No specific involvement*: the intervention had the intention of improving antimicrobial stewardship, but there was no indication that an ASP team was involved in the design or deliver of the intervention.  *ASP team informed or involved*: An ASP team was involved in the design or deliver of the intervention but not the primary driver. These included studies that involved input from multiple departments or where people of different clinical expertise collaborated. This also included QI projects targeting specific diseases where better antimicrobial stewardship practices were not the main focus but were incorporated and supported by an ASP team.  *ASP team led/driven*: The intervention was designed and/or implemented by the ASP team. This included papers studying the effectiveness of new ASP teams.  *Not addressed in the paper*: The paper did not specifically describe the strategy team or it was unclear to reviewers who was involved in the strategy. |
| **Who were the ASP Team Members?** | These individuals were specifically mentioned as being part of the ASP team. Individuals collaborating with the ASP team but not designated as a team member were not included (e.g. microbiologists involved in a testing strategy but were not institutionalized as part of the ASP team).  Some individuals were categorized in multiple roles (e.g. RNs who were described as infection preventionists or MD's who also were described as an administrator, which was captured in the "other" category).  ASP team was involved in the development but another team conducted/delivered the intervention: |
| **Focus of Intervention** | *Patient focused:* Interventions where patients received stewardship education or when clinicians engaged patients in shared decision-making around antimicrobial treatments.  *Clinician focused*: Interventions instituted at the unit level but intended to change individual provider behavior.  *Unit*: Interventions designed to change how the system functions within a particular setting. |
| **Implementation Science Outcomes** | These were evaluations within the study designed to understand the extent to which an intervention was adopted into practice, a strategy influenced perceptions, the actual adoption of a practice, or better use of a resources.  *Knowledge*: Measures the knowledge individuals possess of the intervention or practice being implemented.  *Awareness:* Measures awareness of protocol changes or new guidelines and practices.  *Feasibility*: Measures a provider's perception of the practicality the intervention/  *Appropriateness*: Measures the provider's perception of how compatible the intervention is to the setting or situation.  *Cost:* Measures the financial or non-financial costs of implementing the intervention.  *Fidelity*: Measures the degree to which providers adhere to new guidelines or protocol.  *Reach*: Measures the penetration or level of institutionalization of an intervention.  *Adaptation*: Measures changes to the intervention to accommodate unique contexts.  *Sustainment:* Measures continuation of an intervention over time. |
| **Resource Utilization Outcomes** | These were outcomes related to the use or consumption of institutional resources.  *Duration:* Measurements related to the length of antimicrobial therapy.  *Drug Choice:* Measurements related to changes in antimicrobial usage initiated to provide more appropriate therapy.  *Timing of Antibiotic:* Measurements related to changes to the frequency of antimicrobial treatment delivery or the amount of time between diagnosis and treatment.  *IV to Oral Switch:* Measuring the conversion of patients from IV antimicrobial therapies to oral.  *Diagnostic Switch:* Measuring conversions to more appropriated or precise antimicrobials based on test results.  *Cost:* Measurements related to the cost of antimicrobial prescribing and treatments.  *Measure of overall antibiotic consumption or amount:* Measurements related to overall consumption of antibiotics at the unit or institutional level.  *Dose of antibiotic:* Measurements related to changes in the amount of antibiotic given at a particular time. |
| **Patient Outcomes** | Outcomes related to the health of patients who had healthcare resources applied to them. These could include global or patient-specific outcomes. |
| **Reported Effects** | *All positive effects*: All outcomes in a category were reported as resulting in beneficial improvements.  *All negative effects*: All outcomes in a category were reported as resulting in harmful effects.  *Mixed effects reported*: A combination positive, negative, or null effects, as described above, were reported. |
